# Relationship between Laser Intensity at the Peripheral Nerve and Inhibitory Effect of Percutaneous Photobiomodulation on Neuronal Firing in a Rat Spinal Dorsal Horn

**DOI:** 10.3390/jcm12155126

**Published:** 2023-08-04

**Authors:** Daisuke Uta, Naoya Ishibashi, Yuki Kawase, Shinichi Tao, Masahito Sawahata, Toshiaki Kume

**Affiliations:** 1 Department of Applied Pharmacology, Faculty of Pharmaceutical Sciences, University of Toyama, Toyama 930-0194, Japan; msawa@pha.u-toyama.ac.jp (M.S.); tkume@pha.u-toyama.ac.jp (T.K.); 2 Department of Applied Pharmacology, Graduate School of Medicine and Pharmaceutical Sciences, University of Toyama, Toyama 930-0194, Japan; d2262301@ems.u-toyama.ac.jp; 3 Biomedical Engineering Laboratories, Teijin Institute for Bio-Medical Research, Teijin Pharma Ltd., Tokyo 191-8512, Japan; y.kawase@teijin.co.jp (Y.K.); s.tao@teijin.co.jp (S.T.)

**Keywords:** electrophysiology, spinal, dorsal horn, lamina II, pain, peripheral nerve, photobiomodulation, low-level laser therapy

## Abstract

Photobiomodulation is an effective treatment for pain. We previously reported that the direct laser irradiation of the exposed sciatic nerve inhibited firing in the rat spinal dorsal horn evoked by mechanical stimulation, corresponding to the noxious stimulus. However, percutaneous laser irradiation is used in clinical practice, and it is unclear whether it can inhibit the firing of the dorsal horn. In this study, we investigated whether the percutaneous laser irradiation of the sciatic nerve inhibits firing. Electrodes were inserted into the lamina II of the dorsal horn, and mechanical stimulation was applied using von Frey filaments (vFFs) with both pre and post laser irradiation. Our findings show that percutaneous laser irradiation inhibited 26.0 g vFF-evoked firing, which corresponded to the noxious stimulus, but did not inhibit 0.6 g and 8.0 g vFF-evoked firing. The post- (15 min after) and pre-irradiation firing ratios were almost the same as those for direct and percutaneous irradiation. A photodiode sensor implanted in the sciatic nerve showed that the power density reaching the sciatic nerve percutaneously was attenuated to approximately 10% of that on the skin. The relationship between the laser intensity reaching the nerve and its effect could be potentially useful for a more appropriate setting of laser conditions in clinical practice.

## 1. Introduction

Photobimodulation (PBM) has various effects, such as analgesic effects [1,2,3], anti-inflammatory effects [4,5], tissue regeneration promoting effecst [6,7,8,9] and wound healing [10,11,12]. Meta-analyses of various pain conditions [13,14,15,16,17] have demonstrated the efficacy and safety of PBM for pain. PBM can be used for both acute and chronic pain [1,18] and has the advantage of being noninvasive and safe [14,19]. A previous study showed that PBM can be as effective as the local anaesthetic lidocaine in treating low back pain [20]. Thus, PBM has the potential to complement or replace pharmaceuticals in the treatment of pain.

Although the mechanism is not fully understood [2,3,4,5,21,22], previous studies using electrophysiological techniques have shown that PBM affects the activity of pain-transmitting nerves [23,24,25,26,27,28,29]. These include a few studies in which neural activity was recorded in the spinal dorsal horn, the entry point for pain signals in the central nervous system [30], but these studies do not mention the Rexed laminae of the spinal dorsal horn [28,29]. We previously reported that the 808 nm laser irradiation of the sciatic nerve, exposed through a skin incision in rats, inhibited neuronal firing in the lamina II of the spinal dorsal horn evoked by mechanical stimulation, corresponding to the noxious stimulus of the peripheral cutaneous receptive field [23]. This study suggested that laser irradiation inhibited the activity of Aδ and/or C fibers [23] because Aδ and C fibers innervate lamina II [30]. However, because the sciatic nerve was exposed and the laser was applied directly [23], it is unknown whether percutaneous irradiation, used in clinical practice, also inhibits neuronal firing in the lamina II of the spinal dorsal horn.

Because lasers are scattered and absorbed by biological tissues [31,32,33], such as skin and muscle tissue, the laser intensity reaching the nerves is expected to be lower with percutaneous irradiation, and the resulting effect may also change. Previous studies reporting percutaneous laser irradiation of the sciatic nerve, although not recorded from the spinal dorsal horn, have shown conflicting results regarding inhibition or excitation, depending on the number of laser sites and energy density [24,25]. Thus, the effect of percutaneous laser irradiation on the sciatic nerve remains unclear.

This study aimed to examine whether percutaneous laser irradiation of the sciatic nerve inhibits firing in the lamina II of the dorsal horn. In addition, to examine the relationship between the laser intensity and efficacy, the laser delivered to the nerve during percutaneous laser irradiation was measured using a photodiode sensor.

## 2. Materials and Methods

All experiments were performed in accordance with the Guiding Principles for the Care and Use of Animals in the Field of Physiological Sciences of the Physiological Society of Japan and were approved by the local Animal Experiment Committees of the University of Toyama (approval No. A2020PHA-12 and A2023PHA-13) and Teijin Pharma Limited (approval No. A23-008, respectively). All experiments were performed in the afternoon between 9:00 a.m. and 19:00 p.m. All rats were randomly assigned to the experimental groups and were tested in sequential order. All efforts were made to minimize animal suffering and the number of animals used in this study.

### 2.1. Animals

We used 11 rats in this experiment. Seven-week-old male Wistar rats (Japan SLC Corporation, Hamamatsu, Japan) were used in all the experiments. The rats were kept under environmental control with a 12 h light/dark cycle (lights on at 7:00 a.m.), a temperature (permissive range) of 23 °C (20–26 °C) and a humidity (permissive range) of 55% (30–60%) with free access to food and water.

### 2.2. In Vivo Extracellular Recordings from Lamina II Neurons

We used 6 rats in this experiment. The methods used for in vivo extracellular recordings are described in detail elsewhere [34,35,36,37]. Briefly, the somatic male Wistar rats were anesthetized using urethane (1.2–1.5 g/kg, administered intraperitoneally). The level of anesthesia was periodically tested by assessing hindlimb withdrawal and corneal reflexes. No additional doses were administered during the experiments. After deeply anesthetizing the rats, the skin over the thoracolumbar region and dorsal aspect of the right hindlimb was shaved. Thoracolumbar vertebroplasty was performed to expose Th11–L4, and the animals were placed in the instrumentation. Under a binocular microscope with ×8 to ×40 magnification, after removing the dura mater and cutting the arachnoid membrane to create a space large enough to accommodate a tungsten microelectrode, the surface of the spinal cord was perfused via a glass pipette at 37 ± 1 °C with 95% O_2_–5% CO_2_ equilibrated Krebs solution (10–15 mL/min) containing the following: 117 mM NaCl, 3.6 mM KCl, 2.5 mM CaCl_2_, 1.2 mM MgCl_2_, 1.2 mM NaH_2_PO_4_, 11 mM dextrose and 25 mM NaHCO_3_. Single-unit extracellular recordings from spinal dorsal horn (lamina II) neurons were performed as follows: the electrode was advanced into the spinal dorsal horn (lamina II) neurons at an angle of 30 deg. Recordings were performed from superficial dorsal horn neurons at a depth of 20–150 µm from the surface. The unit signals were acquired using an amplifier (EX1; Dagan Corporation, Minneapolis, MN, USA). The data were digitized using an analog-to-digital converter (Digidata 1400A; Molecular Devices, Union City, CA, USA) and analyzed. To determine the site of stimulation, we searched for sites at which tactile stimulation of the skin (debrided cotton) or unpleasant plucking stimuli (forceps) elicited neural responses. For mechanical stimulation, the skin was bent with thin von Frey filaments, and bending forces of 5.88, 78.4 and 255 mN (0.6, 8.0 and 26.0 g) were applied. Stimulation was applied at the maximum response point of each receptive field of the ipsilateral hind limb for 10 s [38,39,40].

### 2.3. Laser Irradiation

A semiconductor laser source (ML6500 system; Modulight Corporation, Tampere, Finland) was used. The laser light was guided from the laser source with an optical fiber (M28L05; Thorlabs Incorporated, Newton, NJ, USA) and collimated using a lens (SLB-15-30-PIR1; SIGMAKOKI Company, Limited, Tokyo, Japan). The laser power, irradiation time and oscillation mode were controlled using laser source software (ML6700 Controller; Modulight Corporation, Tampere, Finland). The laser power was measured using a power meter (display; NOVAII, sensor; 10A-1.1V; Ophir Japan Limited, Saitama, Japan). The laser conditions are listed in Table 1. The laser was percutaneously applied to the right sciatic nerve.

### 2.4. Measurement of Power Density at the Sciatic Nerve

We used 5 rats in this experiment. The anesthetic solution prepared by mixing Medetomidine (0.4 mg/kg; Nippon Zenyaku Kogyo Company, Limited, Fukushima, Japan), Midazolam (2.0 mg/kg; Sandoz Kabushiki Kaisha, Tokyo, Japan) and Butorphanol (5.0 mg/kg; Meiji Seika Pharma Company, Limited, Tokyo, Japan) was administered subcutaneously to the rats to induce a state of general anesthesia. The right thigh was incised from the ventral side without damaging the dorsal side, and the sciatic nerve was visually identified. Immediately after euthanasia via hyperanesthesia to minimize bleeding, the ventral skin of the thigh was further incised to ensure that the sciatic nerve was visible, and a Si PIN photodiode (S8385; Hamamatsu Photonics Kabushiki Kaisha, Shizuoka, Japan) with a photodetection area of 2 × 2 mm^2^ was implanted at the sciatic nerve. The photodiode was connected to a 100 V/A amplifier, and the voltage was acquired using an oscilloscope (InfiniiVision MSOX4034A; Keysight Technologies Incorporated, Santa Rosa, CA, USA). The laser was used under the same conditions as those for the electrophysiological recordings (Table 1). The photodiode was calibrated such that a voltage of 216 mV corresponded to a power density of 100 mW/cm^2^ at 808 nm. The right paw was sampled, and the distance from the skin surface to the sciatic nerve was measured using a Vernier caliper (CD67-S15PS; Mitutoyo Corporation, Kanagawa, Japan).

### 2.5. Statistical Analysis

Prism v8.4.3 (Graph Pad Software Incorporated, San Diego, CA, USA) was used for the statistical analysis. Data are presented as the mean (SEM). No statistical power calculation was conducted prior to the study, but the sample sizes were based on our previous experience with similar studies, were similar to those generally employed in the field and were selected based on the available data. For statistical analyses, we performed a one-way analysis of variance followed by the Mann–Whitney U test, Wilcoxon matched-pairs signed-rank test or Dunnett’s multiple comparisons; *p* < 0.05 was considered significant. Statistical significance was defined as *p* < 0.05. Randomization methods were not used to assign subjects.

## 3. Results

### 3.1. Effects of Percutaneous PBM on the Neuronal Firing in the Lamina II of Spinal Dorsal Horn Neurons Evoked by Mechanical Stimulation

**Figure 1:**
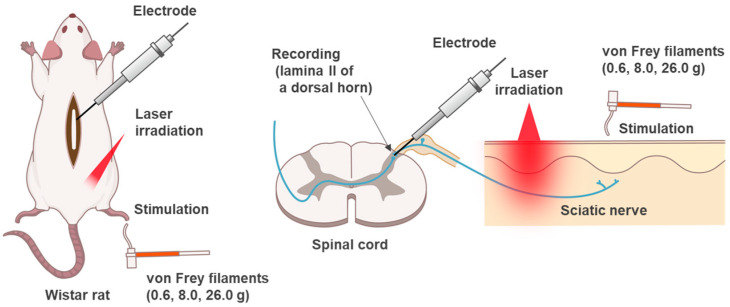
Schematic diagram of the in vivo extracellular recording setup. Recording electrodes were inserted into the lamina II of a rat spinal dorsal horn. The laser was applied percutaneously to the sciatic nerve. Mechanical stimulation with von Frey filaments was applied to the cutaneous receptive field.

The evaluation system shown in the schematic diagram was used to record neuronal firing in the lamina II of the dorsal horn (Figure 1).

**Figure 2:**
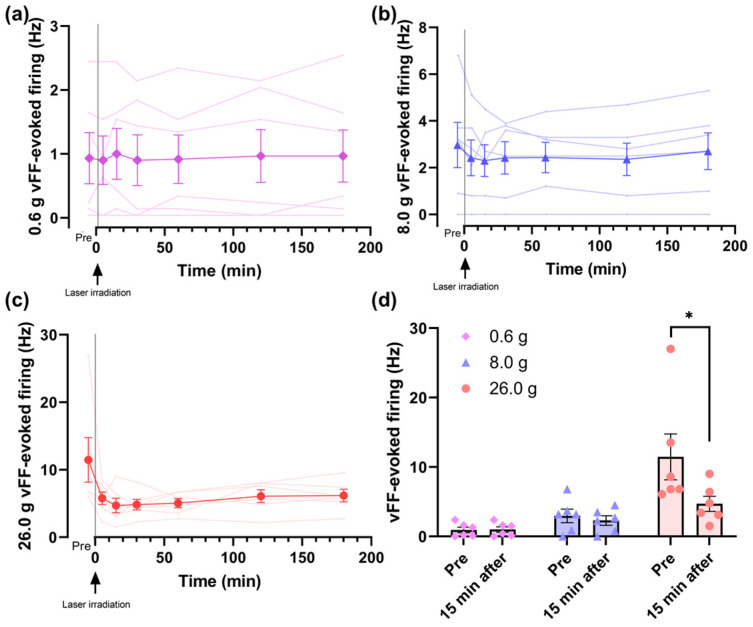
vFF-evoked firing frequency between post and pre-irradiation: (**a**,**b**) Laser irradiation did not affect 0.6 g and 8.0 g vFF-evoked firing. (**c**) Laser irradiation showed a tendency to inhibit 26.0 g vFF-evoked firing frequency at each time point compared to pre-irradiation. (**d**) A comparison was made between the post (15 min after) and pre-irradiation. A significant difference was observed between the post and pre-irradiation at 26.0 g. Data are presented as means ± SEM; Dunnett’s multiple comparisons test or Wilcoxon matched-pairs signed-rank test was used (*n* = 6); * *p* < 0.05 vs. Pre using Wilcoxon matched-pairs signed-rank test; vFF, von Frey filaments; SEM, standard error of the mean.

The vFF-evoked firing frequency showed no change at 0.6 g and 8.0 g and a decreasing trend at 26.0 g compared to pre-laser irradiation (Figure 2a–c). At 15 min after irradiation, when the frequency was most inhibited at 26.0 g, a significant difference was observed compared to pre-laser irradiation at 26.0 g (Figure 2d).

### 3.2. Comparison of the Effects of Percutaneous PBM and Direct PBM on the Sciatic Nerve

**Figure 3:**
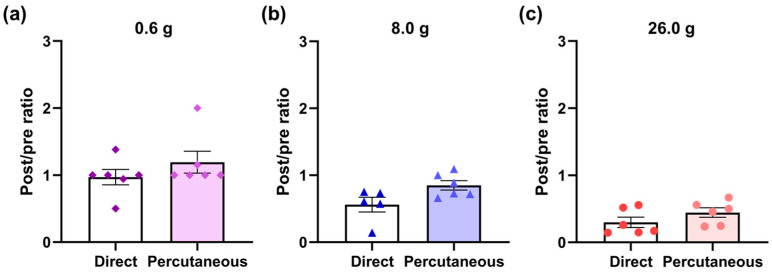
Post- (15 min after) and pre-irradiation ratios. (**a**–**c**) There was no difference between direct irradiation [23] and percutaneous irradiation in post- and pre-irradiation ratios of 0.6, 8.0 and 26.0 g vFF-evoked firing frequencies. Data are presented as means ± SEM; Mann–Whitney U test was used (*n* = 5–6); vFF, von Frey filaments; SEM, standard error of the mean.

We attempted to compare the effects of direct laser irradiation on the sciatic nerve using the same experimental setup in the past [23] with the effects of percutaneous irradiation in this study. The vFF-evoked firing frequency of the neurons varied widely, and it was difficult to compare the firing frequency values. Therefore, we calculated the post-irradiation (15 min later) and pre-irradiation ratios for each neuron. A ratio of 1 means that the firing frequency did not change between post and pre-irradiation, whereas a ratio less than 1 means that the firing frequency decreased at post-irradiation. As a result, there was no difference between direct irradiation [23] and percutaneous irradiation in post- and pre-irradiation ratios of 0.6, 8.0 and 26.0 g vFF-evoked firing frequencies (Figure 3a–c).

### 3.3. Measurement of Power Density at the Sciatic Nerve

**Figure 4:**
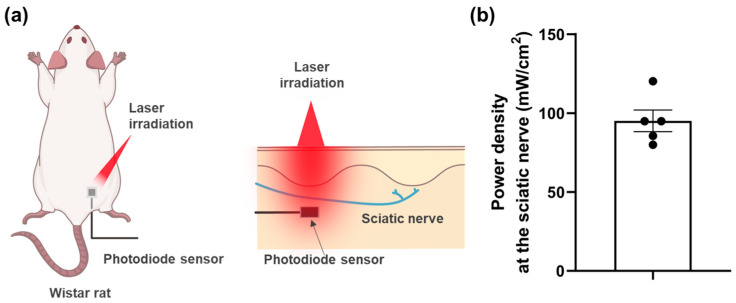
(**a**) Schematic diagram of the experimental setup in which the power density at the sciatic nerve was measured with a photodiode sensor. (**b**) Power density at the sciatic nerve during percutaneous laser irradiation with a photodiode sensor implanted at the right nerve. The power density, which was 1000 mW/cm^2^ at the skin surface, was attenuated to 95.1 ± 6.89 mW/cm^2^ at the sciatic nerve. Data are presented as means ± SEM (*n* = 5); SEM, standard error of the mean.

A photodiode sensor was placed at the sciatic nerve to measure the power density at the nerve during percutaneous laser irradiation (Figure 4a). The power density at the nerve was 95.1 ± 6.89 mW/cm^2^ compared to a power density of 1000 mW/cm^2^ at the skin surface (Figure 4b). The distance from the skin surface to the sciatic nerve was 6.3 ± 0.34 mm.

## 4. Discussion

In this study, we demonstrated that percutaneous laser irradiation of the sciatic nerve inhibited firing in the lamina II of the rat spinal dorsal horn evoked by mechanical stimulation. Among the vFFs used for mechanical stimulation, laser irradiation did not affect 0.6 and 8.0 g vFF-evoked firing but significantly inhibited 26.0 g vFF-evoked firing (Figure 2). The post- (15 min after) and pre-irradiation ratios of vFF-evoked firing frequency did not change between direct and percutaneous irradiation (Figure 3), but the photodiode sensor measurements showed that the power density at the sciatic nerve during percutaneous laser irradiation was attenuated to 9.51% of that on the skin (Figure 4). This study is the first report to show that percutaneous laser irradiation blocked nerve conduction by recording firing in the lamina II of the rat spinal dorsal horn. In addition, this study is also the first report to compare the effects of percutaneous laser irradiation and direct laser irradiation in the same experimental system and to examine the relationship between laser intensity at the nerve and its effects by measuring the actual power density at the nerve during percutaneous irradiation.

Percutaneous laser irradiation of the sciatic nerve significantly reduced the 26.0 g vFF-evoked firing frequency during post-irradiation (15 min after) compared with that during pre-irradiation. Further, the 0.6 and 8.0 g vFF-evoked firing frequencies remained unchanged. This result is consistent with our previous report on the direct laser irradiation of the sciatic nerve [23]. Mechanical stimulation by vFF is equivalent to 26.0 g vFF for a noxious stimulus, 0.6 g vFF for an innocuous stimulus and 8.0 g vFF for an intermediate stimulus [35]. Thus, this study suggests that both percutaneous and direct irradiation inhibit noxious-stimulus-evoked firing. A previous study reported that the direct 830 nm laser irradiation of the peroneal nerve inhibits neural activity in the dorsal root evoked by noxious stimuli, such as pinch, heat and cold stimuli, but not by innocuous stimuli, such as brush stimulation [26]. In this study, we showed the effects of 808 nm laser irradiation on noxious and innocuous mechanical stimuli using different vFFs and found a similar trend to that of a previous study [26]. Our results support the use of PBM for pain treatment in clinical practice, as the firing frequency is also inhibited by percutaneous irradiation, which is used in clinical practice.

According to a previous study to examine the effects of percutaneous laser irradiation by recording from neurons in the rat spinal dorsal horn, percutaneous He–Ne laser irradiation of the peroneal nerve for 30 min significantly inhibited neuronal activity in the spinal dorsal horn induced by formalin administration to the peripheral skin [28]. Although that study does not mention the Rexed laminae of the spinal dorsal horn in which the recordings were made, it is consistent with this study, in that the neuronal activity in the spinal dorsal horn evoked by noxious stimuli was inhibited after laser irradiation.

Although the sham irradiation group was not verified in this study, in a previous report [23], the firing frequency did not change when sham irradiation was performed after an incision of the skin of the right thigh. Because the skin of the right thigh was not incised in the present study, the firing frequency in the sham irradiation group was expected to remain almost the same. Therefore, it is suggested that the results observed in this study were due to percutaneous laser irradiation.

Two previous studies on the percutaneous irradiation of the sciatic nerve, both by the same research group, reported different effects when the number of sites and duration of irradiation varied, even when lasers of the same power were used [24,25]. Specifically, the irradiation of four different sites on the sciatic nerve for 30 s each (225 J/cm^2^ each) at 7.5 W/cm^2^ and 808 nm decreased the amplitude of short-latency somatosensory-evoked potentials (SSEPs) and increased the latency of compound muscle action potentials [24], whereas irradiation at one site for 120 s (900 J/cm^2^) increased SSEPs and left the compound muscle action potentials unchanged [25]. In contrast, when the sciatic nerve site was irradiated at 808 nm and 1 W/cm^2^ for 180 s (180 J/cm^2^), either directly [23] or percutaneously (this study), noxious-stimulus-evoked firing was inhibited. Although our report may contradict previous reports [24,25], the 10–12-week-old rats used in previous reports [24,25] were larger than the 7-week-old rats that we used, and therefore, it is possible that the laser intensity at the sciatic nerve in previous reports [24,25] was lower than that at the sciatic nerve in this study. To accurately validate the relationship between laser intensity and efficacy, it would be desirable to report the laser intensity, such as power density and energy density, at target tissues, such as the sciatic nerve, as in this study. Few studies have investigated the relationship between laser intensity in target tissues and the effect of percutaneous laser irradiation; however, in a previous report, laser irradiation with a power of 10 W and a diameter of 4 cm resulted in a power density of 270 mW/cm^2^ near the L5 dorsal root ganglion (DRG) in rats and improved hyperalgesia with an irradiation time of 120 s (energy density: 32.4 J/cm^2^) [41]. In the present study, the power density at the sciatic nerve was 95.1 mW/cm^2^, so the energy density was 17.1 J/cm^2^ with an irradiation time of 180 s. The energy density at the sciatic nerve in this study was of the same order as the energy density at the L5 DRG in the previous report [41].

To the best of our knowledge, no previous studies have investigated both percutaneous and direct irradiation using the same experimental setup. The post- (15 min after) and pre-irradiation ratios showed no significant differences between the percutaneous and direct irradiation groups (Figure 3). In contrast, with percutaneous irradiation, the power density at the sciatic nerve was approximately 10% lower than that on the skin (Figure 4). These results suggest that the nerve conduction blocking effect of PBM may be equivalent over a relatively wide range of laser intensities, such as power density and energy density. The laser intensities, such as power, power density, energy and energy density, are important parameters of laser characteristics; however, previous reports have not provided a consistent view of the relationship with efficacy. The World Association for Photobiomodulation Therapy recommends an average power of 5–500 mW and an irradiation time of 20–300 s when using 780–860 nm lasers for treatment [42]. There are some reports of the biphasic dose response of PBM [43,44], and there are many studies in which laser parameters are not properly described, making it difficult to consider the relationship between parameters and efficacy [1]. Further, in a previous report, no parameters were found to be associated with efficacy in the treatment of neuromuscular diseases [45]. One of the reasons for the complexity of this discussion is that the laser irradiation conditions on the skin were compared across studies without considering laser attenuation in the skin. Lasers get scattered and absorbed by the skin tissue [31,32,33], and some reports have shown that the laser intensity attenuates exponentially with increasing distance from the skin surface [46,47]. Even for the same disease, the depth from the skin surface to the target tissue can differ depending on the irradiation site, and the laser intensity in the target tissue can also differ. Measuring the laser intensity in the target tissue (such as the sciatic nerve in this study) and understanding its relationship with efficacy can help set appropriate laser conditions. Although it is difficult to implant sensors in humans in clinical trials, it may be useful to investigate irradiation conditions to achieve the desired laser intensity in human target tissues, for example, using light propagation simulations [48,49,50].

The limitation of this study is described. This study suggests that the effects might be equivalent over a relatively wide range of laser intensities. However, this study discusses only two conditions of laser intensity at the nerve. Future studies examining multiple laser intensity conditions could reveal a relationship between laser intensity and efficacy that is not yet clear.

## 5. Conclusions

Percutaneous laser irradiation of the sciatic nerve axon, one of the conduction pathways of a noxious stimulus, with an 808 nm laser inhibited noxious-stimulus-evoked neuronal firing in the lamina II of the spinal dorsal horn. In the case of percutaneous irradiation, the power density at the sciatic nerve was reduced to approximately 10% of that on the skin, but the post- (15 min after) and pre-irradiation ratios did not differ between direct and percutaneous irradiation. Further studies to understand the relationship between laser intensity and efficacy may help establish more appropriate laser conditions in clinical practice.

## Figures and Tables

**Table 1:** Laser parameters.

Wavelength	808 nm
Power	790 mW
Area	0.79 cm^2^
Power density	1 W/cm^2^
Irradiation time	180 s
Energy density	180 J/cm^2^
Mode	Continuous wave
Number of laser irradiations	Once

## Data Availability

The original contributions presented in this study are included in the article, and further inquiries can be directed to the corresponding author.
